# Concerns of Organic Contamination for Sample Return Space Missions

**DOI:** 10.1007/s11214-020-00678-7

**Published:** 2020-05-12

**Authors:** Queenie Hoi Shan Chan, Rhonda Stroud, Zita Martins, Hikaru Yabuta

**Affiliations:** 1 Planetary and Space Sciences, School of Physical Sciences, The Open University, Walton Hall, Milton Keynes, MK7 6AA UK; 2 Present Address: Department of Earth Sciences, Royal Holloway University of London, Egham Surrey, TW20 0EX UK; 3 Code 6360, Naval Research Laboratory, Washington, DC 20375 USA; 4 Centro de Química Estrutural, Departamento de Engenharia Química, Instituto Superior Técnico (IST), Universidade de Lisboa, Avenida Rovisco Pais 1, 1049-001 Lisbon, Portugal; 5 Department of Earth and Planetary Systems Science, Hiroshima University, 1-3-1 Kagamiyama, Hiroshima, 739-8526 Japan

**Keywords:** Sample return, Contamination, Organic matter, Comets, Asteroids, Extra-terrestrial samples

## Abstract

Analysis of organic matter has been one of the major motivations behind solar system exploration missions. It addresses questions related to the organic inventory of our solar system and its implication for the origin of life on Earth. Sample return missions aim at returning scientifically valuable samples from target celestial bodies to Earth. By analysing the samples with the use of state-of-the-art analytical techniques in laboratories here on Earth, researchers can address extremely complicated aspects of extra-terrestrial organic matter. This level of detailed sample characterisation provides the range and depth in organic analysis that are restricted in spacecraft-based exploration missions, due to the limitations of the on-board *in-situ* instrumentation capabilities. So far, there are four completed and in-process sample return missions with an explicit mandate to collect organic matter: Stardust and OSIRIS-REx missions of NASA, and Hayabusa and Hayabusa2 missions of JAXA. Regardless of the target body, all sample return missions dedicate to minimise terrestrial organic contamination of the returned samples, by applying various degrees or strategies of organic contamination mitigation methods. Despite the dedicated efforts in the design and execution of contamination control, it is impossible to completely eliminate sources of organic contamination. This paper aims at providing an overview of the successes and lessons learned with regards to the identification of indigenous organic matter of the returned samples vs terrestrial contamination.

## Introduction

Better understanding of the types of organic matter in the early solar system, and its subsequent evolution, is key to addressing the origin of life on Earth, and to its potential detection elsewhere in the solar system or beyond. Among the fundamental questions that remain only partially answered or controversial are: What is the inventory of organic compounds on comets and asteroids? How much variation among comets and asteroids is there, and how much between comets and asteroids? What is the formation and processing history for the individual components? Are there identifiable features associated with organics from different locations in protoplanetary disks? Are there identifiable features that can be linked to interstellar medium/molecular cloud chemistry? What organics exist in asteroids and comets that may have been delivered to the early Earth? What mixture of asteroidal and cometary material is responsible for the development of life on Earth? Which organic matter is present on other evolved bodies, such as Mars and its moons (Phobos and Deimos), Titan or Enceladus, and how does it relate to those present in small bodies?

**Fig. 1:**
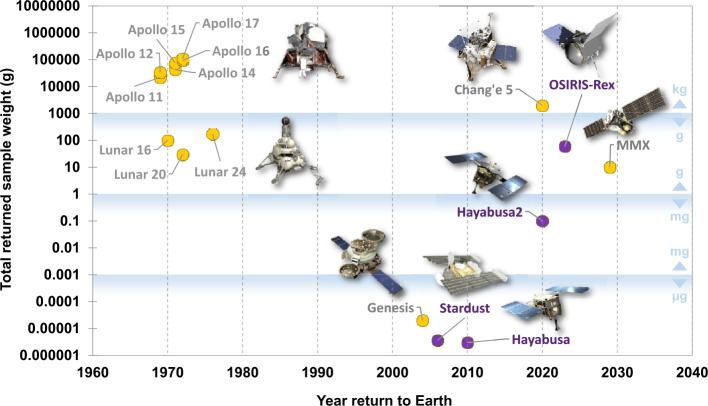
Sample return missions and the total weight (or target weight) of the returned samples. Missions with an explicit mandate to collect organic matter are shown as purple symbols. Minimum target total weights of returned samples are shown in the figure for Hayabusa2 (target: 0.1–10 g) and OSIRIS-Rex (target: 0.06–2 kg). Image credit: NASA, JAXA, CNSA

Sample return missions are essential to fully answer these questions (Fig. 1). Spacecraft-based missions alone, while extremely valuable scientifically in addressing these topics, can only provide partial answers. This is largely because limitations on the *in-situ* instrumentation capabilities, based on payload weight and size, and operating power constraints, are inevitable. With the right returned samples, and the analytical capabilities of laboratories world-wide, researchers can answer extremely complicated, and subtle aspects regarding organic matter, inaccessible with on-board instrumentation. With careful curation, returned samples also remain available in pristine condition for decades as analytical instrumentation continues to improve, new contextual information from other investigations becomes available, and as new questions emerge over time.

Determination of the “right” solar system samples, and how to capture, return, curate and perform organic analysis on them is no simple matter. So far, there have been two completed sample return missions with an explicit mandate to collect organic matter: the NASA Stardust mission to comet 81P/Wild 2; and the JAXA Hayabusa mission to asteroid 25143 Itokawa. Samples from two more asteroids, 162173 Ryugu and 101955 Bennu, are scheduled for return by the JAXA Hayabusa2 and NASA OSIRIS-REx spacecrafts by 2020 and 2023, respectively. Although the CAESAR mission was not selected under the NASA’s New Frontiers Program, it remains as a well-developed comet nucleus sample return mission concept. The first mission that targeted to return up to 200 g of soil from the Martian moon Phobos by 2014 was Russia’s Phobos-Grunt mission (Marov et al. 2004), however the mission terminated in 2012 upon a malfunction that stranded the spacecraft in Earth orbit. A JAXA-led sample return from the Martian moons – the Martian Moons eXploration (MMX) mission, is in the planning stages, which targets to return samples to Earth from Phobos, with a possible return in 2029 (Krüger et al. 2019; Kuramoto et al. 2018; Usui et al. 2020). Analysis of organic matter is also a major motivation behind sample return mission concepts for Mars, Enceladus, and Titan, among others (e.g., Barucci et al. 2012; Mattingly and May 2011; Tsou et al. 2012).

Small bodies, e.g., comets and asteroids, were logical target choices for the first organic sample return due to both scientific and logistical reasons. The planetary protection issues associated with introducing extra-terrestrial organic matter to Earth are rendered moot for asteroids and comet targets, due to the large amounts of such material entering the terrestrial atmosphere daily in the form of interplanetary dust particles (IDPs) and meteorites. Furthermore, to date no such samples have shown to contain any traces of life. The degree of risk associated with Martian sample return however, remains a matter of significant debate. The potential risk of contaminating the Earth with potential extra-terrestrial life forms, combined with the cost of both the return phase of the mission and the appropriate curation methods, has so-far prevented realisation of the long-anticipated Mars sample return. Compared to larger outer body sample return missions, many comet and asteroid targets have the advantage of shorter mission timelines, which generally correlates with reduced cost. Most importantly, if the questions of greatest interest concern tracing the earliest history of organic matter in the solar system and the delivery of organic matter to the early Earth, comets and asteroids are the best sources of the relevant materials.

In all cases, regardless of the target body or planetary protection considerations, minimisation of any terrestrial organic contamination of the returned samples is both a top priority and a major challenge. Each of the completed and in-progress missions has contended with organic contamination mitigation to varying degrees and with different strategies, which provide an important knowledge base for future mission planning. The primary goal of this paper is to provide an overview of the successes and lessons learned with regards to returned organic samples.

## Extra-Terrestrial Organic Matter

The language used to discuss solar system organics varies significantly across the different planetary science communities, so it is important to define explicitly the terms for our discussion. The terms labile, volatile, and refractory for example, take on different meanings depending on the temperature and pressure context. Meteoriticists often draw the distinction between solvent-insoluble organic matter (IOM) and solvent-soluble organic matter (SOM), because the majority of organic matter in meteorites is in the form of complex macromolecules. The IOM is a kerogen-like polymer that is solid at 275K, and thus can be considered non-labile, or refractory (e.g., Alexander et al. 2007; Bonal et al. 2007; Bonal et al. 2006; Busemann et al. 2007; Chan et al. 2017; Clemett et al. 1993; Cody and Alexander 2005; Cody et al. 2008b; Derenne and Robert 2010; Glavin et al. 2018; Kebukawa et al. 2019; Kebukawa et al. 2017; Nakamura 2005; Yabuta et al. 2010; Yabuta et al. 2005; Yabuta et al. 2007). Soluble organic matter in meteorites consists of smaller macromolecules, as well as simpler species such as amino acids, alcohols and sugars. These species may diffuse rapidly through pores in solid samples, and undergo chemical reactions with aqueous fluids, at temperatures as low as 275K, depending on the molecule (e.g., Aponte et al. 2015; Burton et al. 2012; Burton et al. 2013; Burton et al. 2015; Chan et al. 2018b; Elsila et al. 2016a; Elsila et al. 2013; Glavin et al. 2018; Glavin et al. 2010a; Glavin et al. 1999; Glavin et al. 2010b; Glavin and Dworkin 2009; Glavin et al. 2012; Martins 2018; Martins 2019; Martins and Sephton 2009; Pizzarello et al. 2006; Pizzarello and Groy 2011; Pizzarello and Holmes 2009; Pizzarello et al. 2001). In this chapter, we refer to these as labile components, although in the context of comet surfaces, these species are relatively inert. Comets also contain many organic molecules as trapped species in organic and water ices. These we will refer to as volatiles (e.g., methane CH_4_, formaldehyde CH_2_O, methanol CH_3_OH, ammonia NH_3_, hydrogen cyanide HCN, hydrogen sulphide H_2_S (e.g., Bajt et al. 2009; Bockelée-Morvan et al. 2004; Le Roy et al. 2015; Quirico et al. 2016; Sandford et al. 2006)).

## Missions with an Explicit Mandate to Collect Organic Matter

In 2006, the Stardust mission returned the first samples unambiguously identified as originating from a specific comet – comet 81P/Wild 2 (Brownlee et al. 2006; Sandford et al. 2006; Zolensky et al. 2006) (Fig. 1). The Stardust cometary dust collector consisted of an aluminium (Al) frame filled with an array of silica aerogel tiles and Al foils. The tray was exposed to the comet 81P/Wild 2 dust stream at an encounter distance of 237 km at closest approach, and relative velocity of 6.1 km/s (Brownlee et al. 2006; Tsou et al. 2003). The aerogel provided a low density, porous target in which the effects of the hypervelocity capture on the cometary materials could be reduced to the extent that some organic matter was preserved. After the initial impact into the aerogel tile face, the captured cometary particles gradually decelerated by impacting into the nanoscale, amorphous-silica-mesoparticle aerogel backbone, leaving a physical track that is visible to inspection with an optical microscope. Three primary track shapes (A, carrot; B, bulb; and C, hedgehog) were produced (Hörz et al. 2006). Type A tracks are formed when the impacting dust grain retains the majority of its original mass in one or two “terminal” particles deposited at the track terminus. Types B and C are associated with the explosive disaggregation of the impacting dust grains, for which the impacting mass is spread across the track walls, and for the Type C’s, along and at the termini of side tracks. Dust grain components volatised during the heat of impact (labile organics, noble gasses, and possibly ice or water from hydrated dust components) diffused out from the track through the aerogel pore network, eventually adsorbing onto the surrounding silica mesoparticles, and occasionally reaching the Al foils that separate the individual tiles from the collector tray frame (Bechtel et al. 2014; Sandford et al. 2006; Sandford et al. 2010). During the Stardust preliminary examination, organic matter was identified that chemically and structurally resembled the organic matter previously analysed in carbonaceous chondrites, and in anhydrous IDPs thought to have a cometary origin. Some of this material was found to have ^15^N and/or D-rich isotope compositions indicative of formation in cold environment such as the outer solar system, or proto-solar molecular cloud (Aikawa and Herbst 2001; Dartois et al. 2005; Messenger et al. 2003; Pizzarello and Holmes 2009; Remusat et al. 2009; Terzieva and Herbst 2000). Subsequent studies have revealed additional important results, such as the presence of glycine (Elsila et al. 2009). The Stardust mission results provide important context for interpreting results from the Rosetta cometary mission to explore and land on comet 67P/Churyumov–Gerasimenko. However, Stardust was not designed to directly sample the comet surface, or to return any volatiles, which remain important goals for future sample return missions.

The Hayabusa mission targeted the near-Earth S-type asteroid 25143 Itokawa, with the goal of providing the first samples from a known asteroid available for direct laboratory analysis. The Hayabusa sample canister contained two separate chambers (A and B), for collection of dust grains during two distinct touch-and-go encounters (Nakamura et al. 2011; Yada et al. 2014b). According the original sampling plan, a bullet was to be fired into the asteroid surface at each touch down site, so that the sampler horn could funnel the cloud of regolith particles accelerated by the bullet impact into the sample canister. Although the bullets did not fire according to plan, and the number of collection events was reduced to one, dust grains were collected on the walls of the sample canister during a touchdown in 2005 (Yada et al. 2014b; Yano et al. 2006). After the return of the samples to Earth in 2010, more than 2000 individual grains were identified and underwent preliminary analyses. The initial mineralogical and isotopic analyses demonstrated that the Itokawa grains showed a direct affinity to LL-type ordinary chondrites (OCs) (Nakamura et al. 2011; Yurimoto et al. 2011). This result provided the first direct link between S-type asteroids and LL chondrites. The majority (∼90%) of the particles exhibited characteristics of a high degree of thermal equilibration (up to 800°C), consistent with silicate minerals from LL5 to LL6 chondrites (Nakamura et al. 2011). The remaining 10%, appear to less equilibrated, comparable to those found in less equilibrated LL4 chondrites. Because Itokawa is an airless body, and the Hayabusa samples were collected from the surface, the returned grains provide an important source of information about space weathering processes in a regime where ion irradiation from solar wind and galactic cosmic rays dominate (Bonal et al. 2015; Nagao et al. 2011; Noguchi et al. 2011; Tsuchiyama et al. 2011). The combination of thermal metamorphism and radiation processing are unfavourable conditions for the survival of all but the most refractory organic matter. Thus, it is completely consistent that the results of the preliminary search for an SOM component with amino acid analysis and time-of-flight secondary ion mass spectrometry (ToF-SIMS) generated a null result, with an upper limit of a concentration of < 1 parts per million (ppm) estimated from analytical limits (Naraoka et al. 2015). Coordinated H, C, and N isotope measurements, scanning transmission x-ray microscopy (STXM) using x-ray absorption near-edge structure (XANES) spectroscopy and transmission electron microscope (TEM) analysis of a few Itokawa particles of unknown origin (“category 3”) have been performed (Ito et al. 2014; Kitajima et al. 2015; Uesugi et al. 2014; Yabuta et al. 2014a). Although it was challenging to determine their sources only by organic isotopic and molecular analyses, detection of inorganic inclusions (e.g., NaCl, TiO2) as well as comparison with witness coupons led to a conclusion that the possible origins for the category 3 particles include terrestrial contamination.

Two asteroid sample return missions are currently in progress (Fig. 1), this time targeting carbon-rich asteroids. The JAXA Hayabusa2 mission seeks to return samples from the C-type asteroid Ryugu, with a planned return of samples to Earth in 2020 (Tachibana et al. 2014). The NASA OSIRIS-REx plans to return samples from type B asteroid Bennu in 2023 (Barnouin et al. 2019; Hamilton et al. 2019; Lauretta et al. 2017; Lauretta et al. 2015; Walsh et al. 2019). Both asteroids are thought to be related to the class of meteorites known as carbonaceous chondrites, which contain up to ∼3.5 wt% organic carbon (Alexander et al. 2017; Alexander et al. 2007), of which ∼90% is in acid-insoluble, macromolecule polymer form, with the remainder mostly in labile, soluble organic form (Martins 2019; Martins and Sephton 2009; Pearson et al. 2006; Sears and Dodd 1988). Thus, returned samples from these two targets should provide ample organic matter for laboratory analyses designed to address questions regarding the diversity of the organic matter delivered to the surface of the early Earth. Hayabusa2 has taken a sampling approach similar to that of Hayabusa (Sawada et al. 2017), during touch-and-go encounters. In order to obtain samples from a depth expected to be shielded from space weathering processes that would heavily alter the distribution of organic material, a more complex manoeuvre was planned to excavate subsurface material of Ryugu by dropping an explosive small carry-on impactor (SCI) (Arakawa et al. 2020). The SCI was detonated after the spacecraft was deployed to the opposite side of Ryugu for protection against the resultant ejecta. The SCI operation has generated an ejecta curtain and left a crater with a diameter of 20 m on the surface of Ryugu. After the literal settling of the dust, the spacecraft returned to sample the freshly exposed sub-surface regolith in July 2019. The samples will be returned to Earth around the end of 2020. OSIRIS-REx also uses a touch-and-go sampling strategy. In this case, the sample collector will make direct contact with the Bennu surface, and pressurised nitrogen gas will be used to disrupt the surface regolith, driving small pebbles and dust into the collection chamber.

## Determination of Terrestrial Contaminants

Rigorous identification of terrestrial organic contaminants relies on the ability to contrast the types, distribution, structures and isotopic compositions of organic compounds between typical terrestrial contaminants and extra-terrestrial material. A detailed description of the techniques used for studying organic matter in extra-terrestrial material is given in a separate paper of this issue (Martins et al. 2020) so we will not go into details in favour of a brief description here.

The analytical techniques that can be used for studying the organic content of a sample strongly depend on how much of the sample is available for laboratory characterisation. Only very few organic analysis techniques have both the required sensitivity and the high spatial resolution necessary to study the organic matter in small samples, such as the Stardust and Hayabusa missions returned particles. Those that do include two-step laser desorption laser-ionisation mass spectrometry (L^2^MS), Fourier transform infrared spectroscopy (FTIR), Raman spectroscopy, XANES, and NanoSIMS, etc., which are functional group specific, rather than providing characterisation of the entire organic molecule. These techniques focus on refractory organic contents, and can be further classified into: (1) analytical techniques that are spatially-resolved, structure-oriented, and generally non-destructive to the sample (e.g., FTIR, Raman, XANES) and (2) others that are also spatially-resolved but molecule- or isotope-oriented, and yet are destructive techniques as they require the sample materials to be “desorbed” or “sputtered” away in order for them to be detected by the instrument (e.g., L^2^MS, NanoSIMS). The refractory organic contents of extra-terrestrial components typically are highly heterogeneous in elemental and isotopic distributions (e.g., Aléon et al. 2001; Alexander et al. 2013; Chan et al. 2018b; Flynn 2008; Quirico et al. 2005). Organic matter in extra-terrestrial materials displays significant spatial heterogeneity (elemental distribution and ratio) at micrometre-scale (e.g., Alexander et al. 2017; Keller et al. 2004; Quirico et al. 2005). Isotopically, extra-terrestrial refractory organics often contain micrometre-sized D- and ^15^N-rich anomalies (hot spots, $\delta $D value as high as ∼30,000$\permil $ observed for IDPs) (Busemann et al. 2009; Chan et al. 2019b; Keller et al. 2004; Matrajt et al. 2012; Messenger et al. 2008; Messenger et al. 2003; Nakamura-Messenger et al. 2006) mixed with moderately enriched isotopically anomalous organic material. Nevertheless, since these techniques do not provide characterisation of the entire organic molecule, it is impossible to provide a definitive answer as to the direct source of terrestrial contaminant.

Obvious biological contamination in the form of viable fungal and/or bacterial colonies can be readily tested by classical microbiological culture (e.g., Oyama et al. 1970; Regberg et al. 2018). Methodical contamination controls have successfully prevented the returned samples from fungal/bacterial contamination. However, other forms of biological contamination are unavoidable, which can be systematically identified by determining the labile organic components using spectroscopic techniques such as gas chromatography mass spectrometry (e.g., GC-MS, GC/GC-MS, GC-ToF-MS, GC-QqQ-MS etc.) and liquid chromatography (e.g., LC-MS, LC-ToF-MS, LC-FD/QToF-MS etc.). For example, amino acids, monomers of protein, have several characteristics that can be used to identify those that are biotically synthesised: (1) proteins and enzymes are made up of ∼20 major proteinogenic amino acids (e.g., glycine, alanine, valine, aspartic acid, glutamic acid, leucine), while several extra-terrestrial amino acids (e.g., isovaline, $\alpha $-aminoisobutyric acid ($\alpha $-AIB), $\beta $-amino-butyric acid ($\beta $-ABA)) are not used in terrestrial proteins (e.g., Botta and Bada 2002; Burton et al. 2012; Chan et al. 2018b; Cronin and Chang 1993; Ehrenfreund et al. 2001; Elsila et al. 2016a; Glavin et al. 2010b; Martins et al. 2015; Martins and Sephton 2009), (2) proteins for most microorganisms are made of only the L-enantiomer of chiral amino acids, leading to L-homochirality or a significant excess in the L-enantiomers (e.g., Bada 1995; Blackmond 2010; Glavin et al. 1999; Glavin et al. 2012; Herd et al. 2011; Myrgorodska et al. 2015; Pizzarello et al. 2012), (3) terrestrial amino acids are depleted in the heavy isotopes of carbon, hydrogen and nitrogen compared to extra-terrestrial amino acids (e.g., Burton et al. 2013; Chan et al. 2016; Cronin and Chang 1993; Elsila et al. 2016a; Elsila et al. 2012; Martins et al. 2007; Pizzarello et al. 1994; Pizzarello et al. 2004).

## Potential Sources of Contamination

**Fig. 2:**
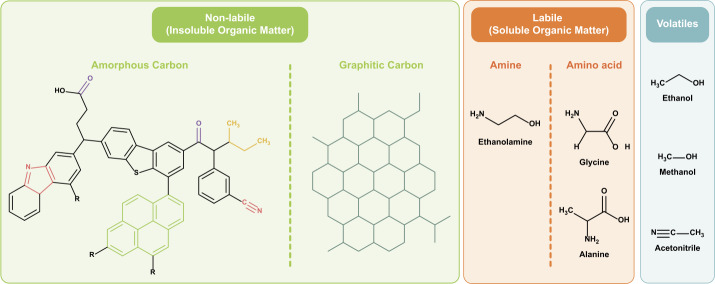
Examples of structures of the organic contaminants described in Table 1

Despite the dedicated efforts in the design and execution of contamination control (Allen et al. 2011; Calaway et al. 2019; Dworkin et al. 2017; McCubbin et al. 2019; Sandford et al. 2010; Yada et al. 2014b), it is impossible to *completely* eliminate sources of organic contamination. The complex nature of a sample return mission elucidates the diversity of potential scenarios where various sources of organic contamination could be introduced into the returned samples (Table 1 and Fig. 2).

**Table 1:**
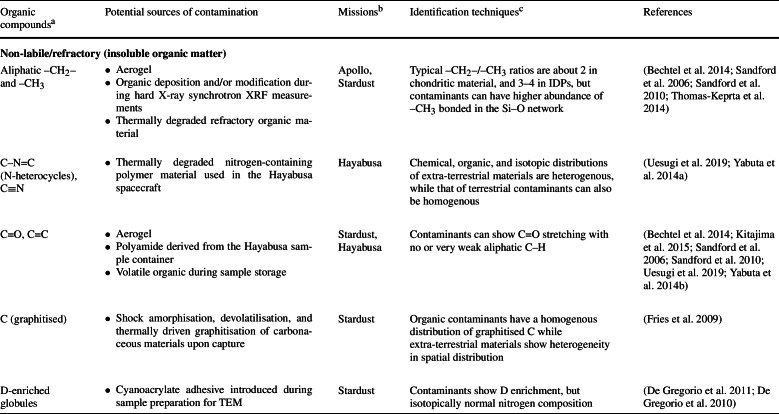

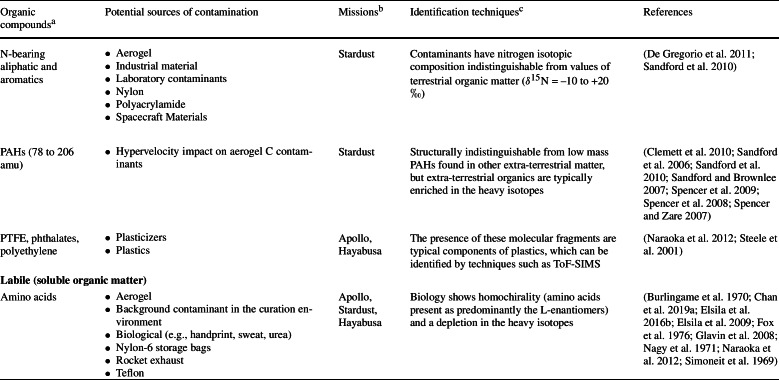

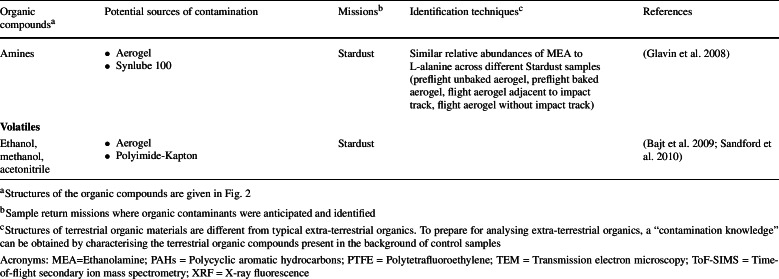
List of terrestrial organic contaminants detected in samples returned by space missions

**Fig. 3:**
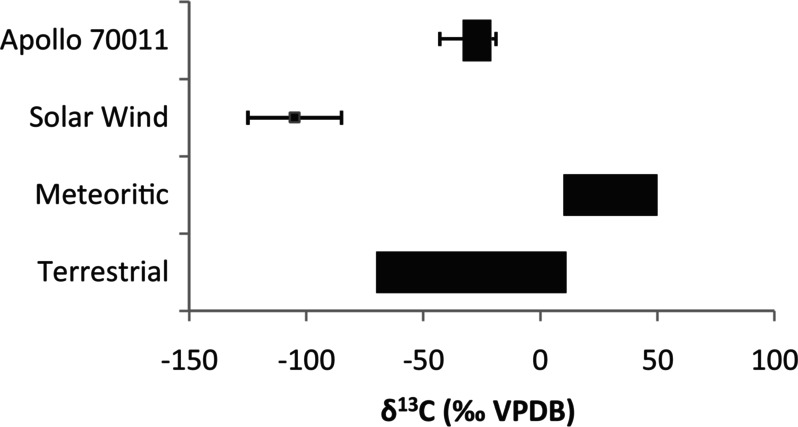
Range of values of $\delta ^{13}$C measured for amino acids in the lunar regolith Apollo 70011, and comparison to the values of solar wind, carbonaceous meteorites and terrestrial sources (Elsila et al. 2016b)^1^

Organic contamination is to some degree inevitable. Contaminants can potentially be accrued during the design and construction of the spacecraft and associated components, on the course of the mission, during the sample recovery and curation processes, and/or during sample characterisation after their dissemination to analytical teams. For example, the lunar samples returned by the Apollo missions were found to contain amino acids derived from terrestrial biological contamination as the $\delta ^{13}$C isotopic values of glycine, $\beta $-alanine, and L-alanine were in the range of terrestrial biological sources, ranging from –21$\permil $ to –33$\permil $ (Elsila et al. 2016b) (Fig. 3).

Some Hayabusa category 3 samples contain carbon-rich organic contaminants that overprinted the extra-terrestrial organic signatures detected by scanning electron microscopy/energy dispersive X-ray spectrometry (SEM/EDS) (Uesugi et al. 2019; Uesugi et al. 2014), Raman spectroscopy (Kitajima et al. 2015), XANES (Yabuta et al. 2014a), NanoSIMS (Ito et al. 2014), and ToF-SIMS (Naraoka et al. 2012). Preflight monitoring of the nitrogen-purged sample cabinet desiccator box by the OSIRIS-REx team also showed a steady increase of volatile compounds although at very small amounts (Dworkin et al. 2017). In this section, previous reports of organic contaminants identified in mission returned samples will be addressed and discussed, based on the organic characterisation of samples returned by the Apollo, Stardust and Hayabusa missions.

### Apollo Missions (1969-1972)

**Fig. 4:**
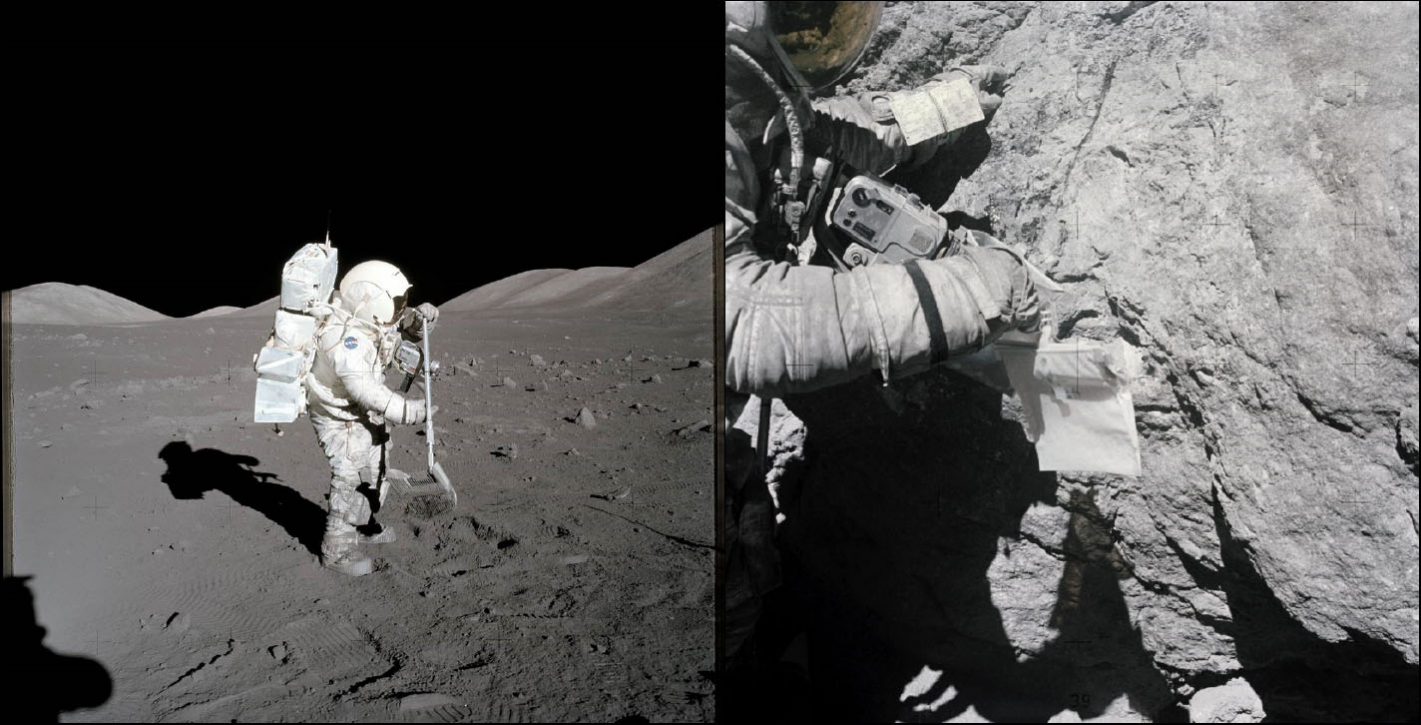
(Left) Astronaut collects lunar rake samples at Station 1 during the Apollo 17 mission’s first spacewalk at the Taurus-Littrow landing site. (Right) During the third Apollo 16 extravehicular activity (EVA), astronaut examines the surface of a boulder at North Ray Crater with a 20-bag dispenser attached to his right wrist (Image credit: NASA)

Apollo 11 marked the first successful sample return from another solar system body. Following the return of Apollo 11 in July 1969, numerous studies have analysed the returned lunar samples in search of organic compounds (e.g., Brinton and Bada 1996; Elsila et al. 2016b; Fox et al. 1976; Glavin et al. 2010c; Hare 1972; Hare et al. 1970; Nagy et al. 1971; Steele et al. 2010; Thomas-Keprta et al. 2014). Lunar samples were collected by astronauts while on traverses, with the use of tools such as scoops, rakes, tongs, and electric drill (Fig. 4), and the samples were stored in separate documented sample bags made of Teflon film reinforced by an aluminium band around the rim (Allton 1989). However, the use of Teflon plastic bag could have contributed to the plastic contamination (fluorinated carbon compound detected by ToF-SIMS) identified in lunar samples (Steele et al. 2001).

In parallel to the search for indigenous organics, immense efforts have been made to address the possible sources of terrestrial contamination that might have influenced the interpretation of the organic compositions of the lunar materials. An early study reported that the Apollo 10 and 11 spacecraft were contaminated with terrestrial bacteria (Puleo et al. 1970), which were probably acquired when the spacecraft were tested and assembled in areas that had more environmental and personnel controls. Fortunately, viable bacteria were not detected in the lunar samples when tested by classical microbiological culture (Holland and Simmonds 1973; Oyama et al. 1970).

Without the technology that could determine the chirality and isotopic compositions of organic compounds, earlier studies could only determine the origin of the organics by comparing that to typical biogenic organics and their distributions. However, this led to divergent conclusions, as some determined that the detected organics were of terrestrial origin (e.g., handprint, urea, and Teflon contamination (Nagy et al. 1971), rocket exhaust (Burlingame et al. 1970; Fox et al. 1976; Simoneit et al. 1969)), while others determined the opposite, that the detected organics were indigenous to the lunar samples (e.g., Harada et al. 1971; Mitchell et al. 1971). A variety of other potential sources of contamination were also noted, such as terrestrial water, exhaust products from the lunar descent engine and reaction control system engines, lunar module outgassing, astronaut spacesuit leakage and venting of life support backpack, particulate abraded from spacesuits, venting of lunar module fuel and oxidiser tanks, cabin, and waste systems (Epstein and Taylor 1972; Simoneit and Flory 1970).

With the availability of the more sensitive high performance liquid chromatography fluorescence detection (HPLC/FD) technique, and based on the amino acid enantiomeric ratio, alanine and aspartic acid detected in Apollo 17 mission returned lunar soil were attributed to terrestrial biogenic contamination accrued during collection, transport, and analysis of the lunar soil (Brinton and Bada 1996). Although the quantities of amino acids in lunar soils made compound specific isotopic measurements challenging at that time, the extremely low D/L enantiomeric ratio of amino acids (D/L < 0.05) was a strong evidence for a biotic origin for the detected amino acids. 30 years after the return of Apollo 11, Kozar et al. (2001) proposed a null hypothesis, that pristine lunar samples should not contain markers (muramic acid and 3-hydroxy fatty acids) for terrestrial bacteria. This null hypothesis was confirmed by gas chromatography-tandem mass spectrometry (GC-MS/MS) analysis, meaning that similar markers detected in pristine lunar samples in the future should be extra-terrestrial. More recent studies in which highly sensitive modern instruments were used have indicated the presence of organic materials, which are composed predominately of amorphous, structurally disordered kerogen-like organic matter that is distinctive from terrestrial contamination (Thomas-Keprta et al. 2014). However, the detected organics were suggested to be contributed by meteoritic delivery to the surfaces of the Moon, instead of synthesised indigenously on the Moon.

In 2016, with the use of a gas chromatography coupled with mass spectrometry and isotope ratio mass spectrometry (GC-MS/IRMS), Elsila et al. (2016b) determined the carbon isotopic compositions and enantiomeric ratios of the amino acids in seven lunar regolith samples. The strong excess of the L-enantiomer of proteinogenic amino acids (aspartic acid, glutamic acid, serine, threonine, and valine), as well as a terrestrial $\delta ^{13}$C isotopic values, led the authors to conclude that terrestrial contamination was the primary source of the majority of the detected amino acids. In agreement with Fox et al. (1976), lunar module exhaust was not the primary source of the observed amino acids (Elsila et al. 2016b). The level of amino acid contamination was higher in non-curated samples compared to samples stored under NASA curation, reflecting terrestrial contamination accrued in non-curatorial laboratory environment for over 40 years. However, the source of the contamination is unknown, except for a potential contamination from Nylon-6 storage bags, as one of the amino acids detected in the lunar soil – $\varepsilon $-amino-n-caproic acid (EACA) – is a monomer released upon hydrolysis of Nylon-6 (Glavin et al. 2006). The presence of Nylon-6 in lunar samples is also supported by the detection of fibrous materials with a m/z 114 fragment on the ToF-SIMS spectra, which is characteristic of the monomer repeat unit of Nylon-6 (Steele et al. 2001).

**Fig. 5:**
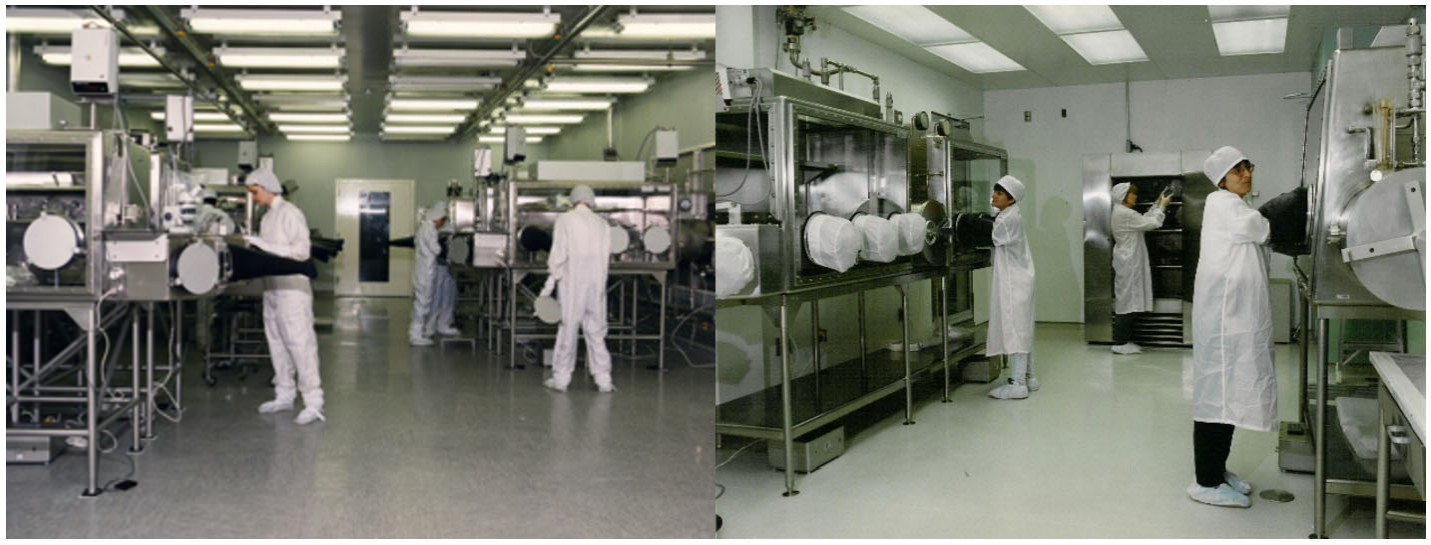
(Left) Lunar Curation Facility and (Right) Antarctic Curation Facility at NASA JSC (Image credit: NASA)

The Apollo samples have been stored under strict isolation conditions in an ISO class 6 clean room for the past 50 years, where all pristine Apollo samples are curated and processed inside gloveboxes filled with inert gaseous nitrogen (Allton et al. 1998). Despite the efforts in contamination control (Allen et al. 2011; Flory and Simoneit 1972), it is possible that terrestrial contamination could have occurred during this extended curation period. Hydrocarbons, plasticizers, solvents, silicones, and rubbers have been detected inside Lunar curation gloveboxes (Calaway et al. 2014), some of which are unavoidable as common plasticizers from outside air could have been released into the laboratory through the air handling system which were not effectively filtered. The concern of contamination of lunar samples during long-term curation was further addressed by Fox (2002). Fox (2002) suggested that the Lunar Curation Facility at Johnson Space Center (JSC) was primarily concerned with keeping the collection clean from chemical contamination but not essentially biological contamination. Similar to the Lunar Curation Facility, the Antarctic Curation Facility at JSC is also an ISO class 6 clean room which has been upgraded from ISO class 7 in 1999–2000 (Allen et al. 2011; Calaway et al. 2019) (Fig. 5). Meteorite samples are either processed inside gaseous nitrogen filled gloveboxes (carbonaceous chondrites and martian meteorites), or on class 100 laminar flow benches (all other types) from which a small amount of viable fungal and bacterial colonies has been observed (4 colony forming units (CFU)/25 cm^2^) (Regberg et al. 2018).

### Stardust Mission (1999-2006)

The Stardust mission has returned to Earth thousands of grains from comet 81P/Wild 2 and interstellar dust. A great effort has been made to identify the possible sources of organic contamination and characterise the potential contaminants by a variety of analytical techniques, such as infrared (IR) microscopy, nuclear magnetic resonance (NMR), STXM-XANES, L^2^MS, liquid chromatography with ultraviolet (UV) fluorescence detection and time-of-flight mass spectrometry (LC-FD/ToF-MS) analyses, etc. (e.g., Sandford et al. 2010). These detailed studies indicated the presence of various sources of organic contaminants, such as intrinsic organic compounds found in silica aerogel that was used to capture the Stardust samples, contaminants introduced during sample analyses, organic material being altered during hypervelocity capture, as well as new organic compounds being synthesised by impact heating.

**Fig. 6:**
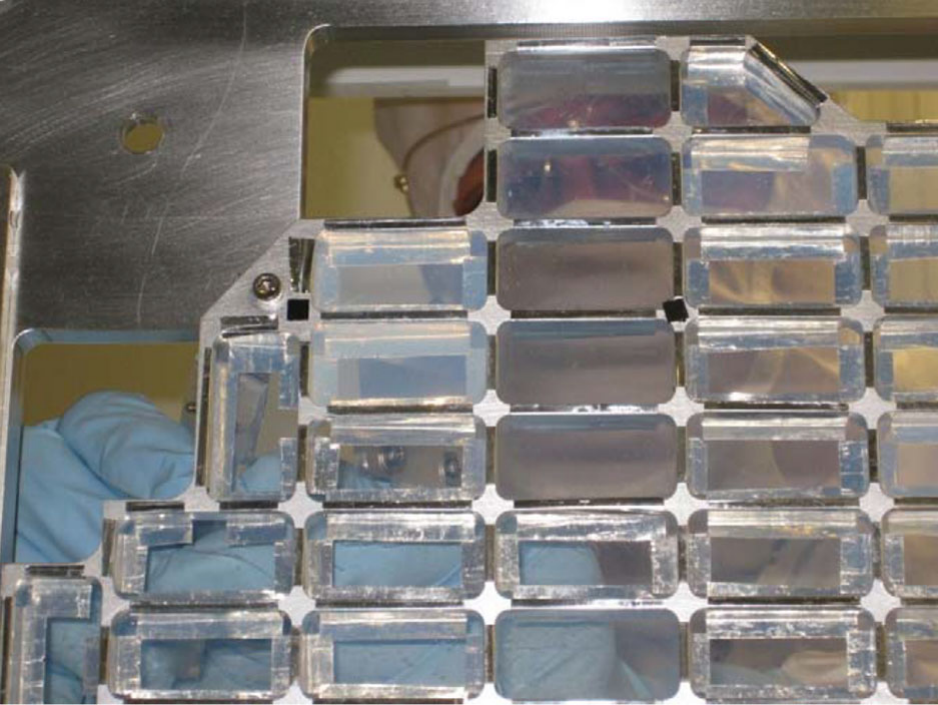
Portion of Stardust interstellar tray showing the aluminium frame, aerogel tiles (4 cm long), and aluminium foil strips wrapping on all four sides of the aerogel tiles (Allen et al. 2011)^2^

The Stardust samples were captured in aerogel tiles, which were wedged into the sample collection trays and wrapped on all four sides with aluminium foil (1100 aluminium) to capture impacting dust particles as well as facilitate aerogel removal (Tsou et al. 2003) (Fig. 6). The aerogel was produced from tetramethyl and/or tetraethyl orthosilicate and the manufacturing process involved the use of ethanol, water, nitric acid, ammonium hydroxide, and acetonitrile (Tillotson and Hrubesh 1992). A silicone-based mould release (Synlube 100) was sprayed into the aerogel tile moulds in order to prevent aerogel from adhering to the moulds during the gelation process. The aerogel should ideally consist solely of Si and O. However, preliminary studies of the original aerogel collector tiles had identified a variety of organic contaminants present at low abundances (Sandford et al. 2006; Sandford et al. 2010).

IR analyses of the original aerogel collector tiles suggested the presence of “native” refractory organic carbon in the forms of Si–CH_2_ and Si–CH_3_ (Bechtel et al. 2014; Sandford et al. 2006; Sandford et al. 2010). The aliphatic –CH_2_– and –CH_3_ groups of the contaminant are covalently bonded to the aerogel network (Tsou et al. 2003), and have IR C–H stretching absorption features in the 3000–2800 cm^−1^ (3.33–3.57 μm) region. The –CH_3_ absorption is significantly stronger than the –CH_2_– absorption, therefore it is easily discernible from typical comet 81P/Wild 2 organics that have high –CH_2_–/–CH_3_ ratios (Keller et al. 2006; Rotundi et al. 2008; Sandford et al. 2006). However, Si–O has a typical stretching absorption feature at around 1100 cm^−1^ (10 μm) that is characteristic of amorphous silicates. The high abundance of Si–O leads to an intensive absorption feature that has saturated the IR spectra. Therefore, it was impossible to determine the –CH_3_/Si–O ratio with the IR technique, and thus the absolute abundance of aliphatic –CH_2_ and –CH_3_ contaminants is unknown.

The impurities in aerogel can also be reflected by Raman spectroscopy. With a porosity of up to 99 vol%, the intensities of the Raman bands of amorphous silica at low wavenumbers (i.e., in the Si–O–Si bending and Si–O stretching region) are extremely weak, and the Raman spectra of pure silica aerogel should be fluorescence-free. However, Stardust aerogel shows very strong Raman peaks at around 2850 cm^−1^ (symmetric CH_2_ stretch), 2908 cm^−1^ (asymmetric CH_2_ stretch), and 2965 cm^−1^ (antisymmetric CH_3_ stretch), which are typical for alkanes (Rotundi et al. 2008; Wopenka 2012). In addition to the alkane features, the Stardust aerogel also has bands in the at ∼ 999 and 1029 cm^−1^ (breathing mode of benzene), as well as in the 1300–1600 cm^−1^ region, which correspond to the presence of CH_2_ (1298 cm^−1^), N=N (1444 cm^−1^), and C=C (1600 cm^−1^) functional groups, and overlap with the graphite band of the sp^2^-bonded carbon feature of amorphous carbon typically observed for extra-terrestrial organics.

Although the absolute abundance of the organic contaminant in the aerogel is unknown, it was possible to estimate the C/Si ratio of the organic carbon in aerogel via NMR analyses (Sandford et al. 2010). NMR analyses indicated that the background C/Si did not exceed 0.12 (or 0.06 in the case of C/O) in the aerogels, which was significantly lower than that observed for organic particles extracted from aerogel coupons (C/Si ≈ 1.7; and C/O ≈ 0.6) (Cody et al. 2008a). The NMR data also indicated that the organic carbon has a very simple chemistry that consists of just a few simple functional groups, which are readily converted into highly volatile species upon heating, e.g., methanol, ethylene, ethanol. This group of organic contaminants can be reduced by subjecting the Stardust samples to heating. However, even though the degree of C contamination could be reduced by <2 wt% by heating to 300 °C for 72 h in a controlled flow of filtered air at 5–6 psi, it could not be completely removed, with the residual organics chiefly in the form of aliphatic –CH_2_– and –CH_3_ (Tsou et al. 2003).

**Fig. 7:**
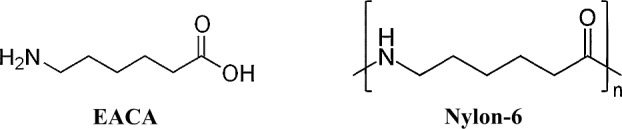
Chemical structures of EACA and Nylon-6

The labile organic contents of Stardust materials (preflight aerogel, Stardust flight aerogel witness tiles, comet-exposed aerogel, comet-exposed foil) have been explored by LC-FD/ToF-MS analysis (Elsila et al. 2009; Glavin et al. 2008; Sandford et al. 2010). Only trace levels of labile organic compounds were identified in the preflight aerogel which included amino acids [L-aspartic acid, L-glutamic acid, L-serine, glycine, $\beta $-alanine (BALA), $\gamma $-amino-butyric acid (GABA), L-alanine, EACA] and amines [ethanolamine (MEA), methylamine (MA), ethylamine (EA)], with a concentration of 0.04 to 3.4 nmol/g (which translates to ∼4 to 340 parts per billion (ppb), with an average molar mass of ∼100 g/mol of all amino acids and amines listed above) (Glavin et al. 2008). EACA and glycine are the two most abundant amino acids detected. D-amino acid was not detected in the preflight aerogel above the 0.1 nmol/g (∼10 ppb) level. The low D/L enantiomeric ratios indicate that most of the amino acids are potentially terrestrial contaminants. The sources of amino acids and MEA detected in Stardust aerogel were suggested to be derived from the aerogel, Nylon-6 storage bag during curation, and Synlube 100 (Table 2). Data from a subsequent carbon isotopic analysis of Stardust comet-exposed foils also confirmed that EACA ($\delta ^{13}$C = −25 ± 2$\permil $) was very likely derived from the Nylon-6 storage and shipping bags used during curation (Elsila et al. 2009) (Fig. 7). Nevertheless, glycine, MA and EA were shown to be indigenous to the comet (Table 2) (Elsila et al. 2009; Glavin et al. 2008). Glycine was suggested to have a cometary origin as it was only detected on the comet-exposed side of the Stardust foil sample which indicated that glycine was not a contaminant from the foil (Glavin et al. 2008). Furthermore, the $\delta ^{13}$C value for glycine of +29 ± 6$\permil $ is well outside the terrestrial range for organic carbon of −6$\permil $ to −40$\permil $ (Elsila et al. 2009), and thus supports an extra-terrestrial origin for glycine. The MA to EA ratio in Stardust comet-exposed materials strongly indicates that these two amines are cometary in origin. The molar ratio of MA to EA in Stardust comet-exposed aerogel and foil (1.0–1.8) is distinct from that in contamination control samples such as preflight aerogel (6.8), SRC heatshield (6.1), and Synlube 100 (0.1) (Glavin et al. 2008). MA and EA are present predominantly in an acid labile bound form, rather than as a free amine, indicating that comet 81P/Wild 2 contains labile amide-rich organic polymer.

**Table 2:** List of major amino acids and amines detected in Stardust materials and their potential source(s) (Elsila et al. 2009; Glavin et al. 2008)

Amine detected	Source
Glycine	Comet 81P/Wild 2
BALA	Aerogel
L-alanine	Aerogel
GABA	Aerogel (partial bakeout)
EACA	Nylon-6 (sample curation)
MEA	Synlube 100, Aerogel
EA	Comet 81P/Wild 2
MA	Comet 81P/Wild 2

**Fig. 8:**
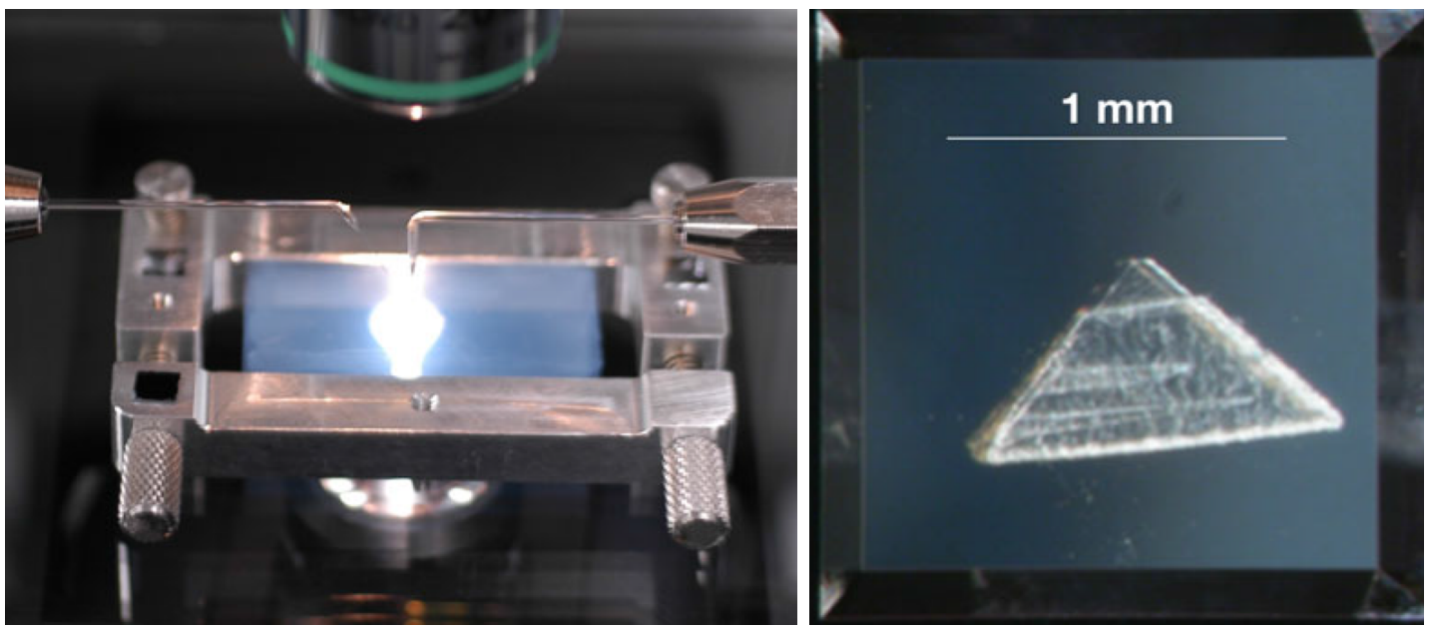
(Left) An aerogel tile mounted in a custom-designed aluminium clamp, with needles that are being prepared to begin cutting a picokeystone. (Right) A picokeystone is mounted between two Si_3_N_4_ windows (Frank et al. 2014).^3^

Impact tracks in the aerogel collector were harvested in wedges of aerogel (typical thickness approximately 500 μm) called “keystones” (∼70 μm thick for “picokeystones”) (Frank et al. 2014; Westphal et al. 2004) (Fig. 8). Characterisation of picokeystones extracted from the Stardust comet and interstellar dust collectors suggested that terrestrial contamination occurred after extraction through the adsorption of volatile organics during sample storage and/or sample transfer (Bechtel et al. 2014). STXM-XANES analysis of 9 picokeystones indicated absorption features around 285 eV and 288.2 eV, which can be assigned to C=C of aromatic carbon and C=O respectively (Sandford et al. 2010). Synchrotron FTIR microscopy analyses revealed organic contamination via beam damage in the form of organic deposition and/or modification during hard X-ray synchrotron X-ray fluorescence (XRF) measurements (Bechtel et al. 2014). In particular, hard XRF analysis can alter the organic content by increasing the –CH_2_–/–CH_3_ ratio significantly.

**Fig. 9:**
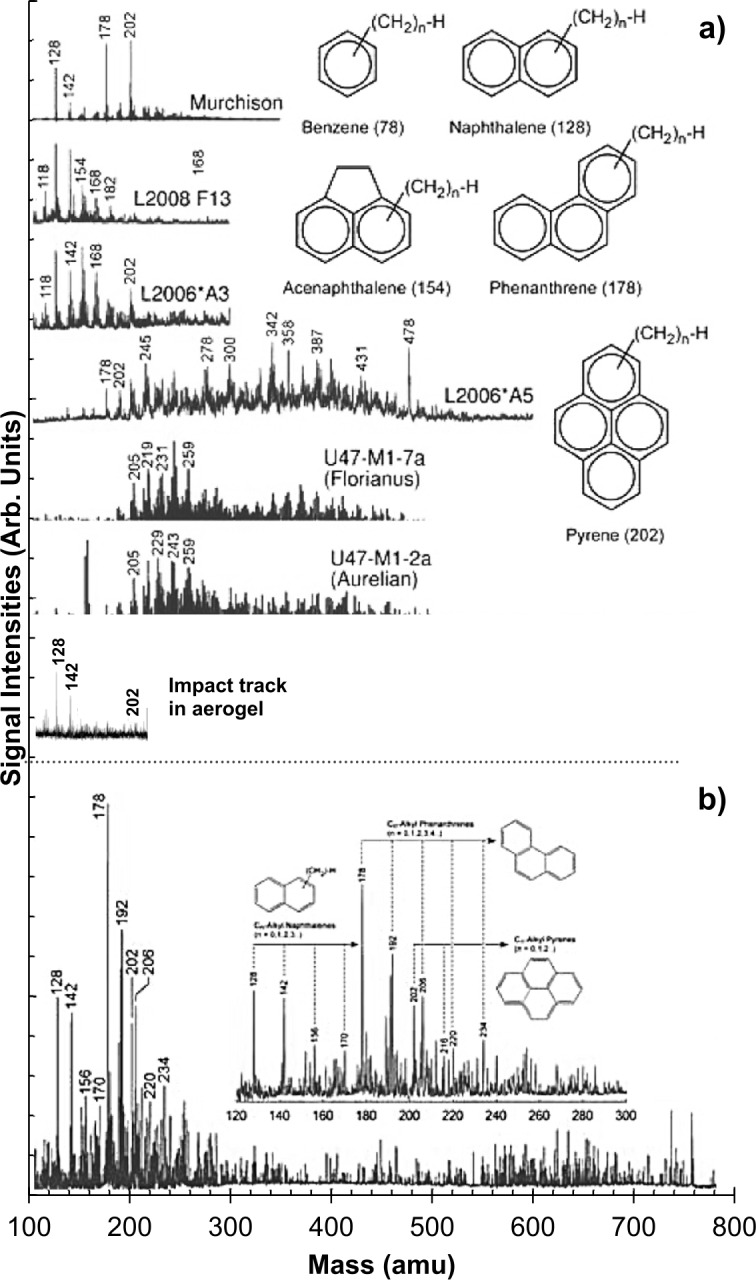
(a) The L^2^MS spectra of the Murchison carbonaceous chondrite, stratospheric IDPs, and a keystone-dissected glass bead impact track in aerogel (Sandford et al. 2006; Spencer et al. 2008). (b) The L^2^MS spectrum of Stardust sample. The PAH distribution of Stardust sample is complex and contains higher molecular weight PAHs, while meteorite, IDPs and impact generated compounds show relatively simple populations, and mostly dominated by small PAHs

In addition to the presence of “native” organics in Stardust aerogel, simulation experiments suggested that organic contaminants could be altered, destroyed, or synthesised during the hypervelocity impact (at approximately 6.12 km/s) of the Stardust particles as they were captured in Stardust aerogel. The simulation experiments were conducted by hypervelocity impact of micrometre-size borosilicate glass beads into Stardust-type aerogel, and IR laser pulse heating of Stardust aerogel (Sandford et al. 2006; Sandford et al. 2010; Sandford and Brownlee 2007; Spencer et al. 2009; Spencer et al. 2008; Spencer and Zare 2007). Based on L^2^MS examinations of the simulation experiment products (keystone-dissected borosilicate glass bead impact tracks in aerogel), a low-intensity distribution of polycyclic aromatic hydrocarbons (PAHs) at the initial bead impact site was observed. The range of low-abundance artefactual PAHs includes toluene (92 atomic mass unit (amu)), styrene (104 amu), naphthalene (128 amu), phenanthrene (178 amu), and pyrene (202 amu) (Spencer et al. 2009; Spencer et al. 2008). Although the L^2^MS technique is particularly well suited for mapping the spatial distribution of PAHs at the micrometre scale and the sub-attomole level (1 amol = 10^−18^ mol) (Clemett et al. 2010; Clemett and Zare 1997), it was shown that a low-mass envelope of aromatic compounds could be synthesised from the original aliphatic carbon in the aerogel by high laser desorption power, which includes the same PAHs as those detected in the IR laser pulse heating simulation experiment (Sandford et al. 2006; Spencer and Zare 2007). Silica aerogels are particularly prone to PAH contamination due to the adsorption properties of SiO_2_ surfaces and their high, density-dependent surface area (Clemett et al. 2010). Fries et al. (2009) has also conducted hypervelocity experiment by firing coal samples into aerogel. It was shown that graphitic materials in direct contact with aerogel could amorphise during aerogel capture, while relatively disordered, heteroatom-rich materials could be devolatilised and then re-condensed within the particle. It is challenging to determine whether the detected PAHs are indigenous to the Stardust samples. Nevertheless, the distributions of the synthesised PAHs in Stardust samples are different from those in the Murchison carbonaceous chondrite and some IDPs (Fig. 9), in particular, the L^2^MS spectrum of Stardust sample is complex and contains higher molecular weight PAHs including alkylated (C_0_–C_4_) phenanthrenes and pyrenes (Sandford et al. 2006).

Transmission electron microscopy analyses of organic matter in capture tracks in the Stardust aerogel collectors have identified extremely N-rich organic matter that is distinctive from typical interstellar organic molecules. Based on C- and N-XANES analyses, this N-rich material was thought to be polymeric contamination, which is aliphatic and dominated by amide (CONHx) functional groups (Cody et al. 2008a), similar to amide polymers such as polyacrylamide and nylon. As these samples also contain nanoscale metal-oxide crystallites, an industrial origin was proposed for their origin (De Gregorio et al. 2011). These N-rich materials could have been present within the aerogel collector during the construction of the Stardust spacecraft, or introduced during particle embedding, although such procedure was conducted in a clean environment and used high-purity sulphur (Cody et al. 2008a; De Gregorio et al. 2011; Wirick et al. 2009).

Other potential sources of contamination to Stardust materials have been explored, which include the landing site soils, the thermal protection system of the sample return capsule (i.e., the pyrolytic C ablator heatshield and backshell), their solid ablation products, and atmospheric vent filter etc. Fortunately, the integrity of the capsule during landing underscores that the collectors were unlikely contaminated by these external sources. The nature of the organic matter detected in these materials differs significantly from the organics seen in the cometary collector, therefore these potential sources did not contribute any significant contaminants to the collectors.

### Hayabusa Mission (2003-2010)

The first mission that brought back to Earth samples from an asteroid was achieved by the JAXA’s Hayabusa spacecraft which visited the near-Earth S-type asteroid (25143) Itokawa (Yada et al. 2014b). Since its return to Earth in 2010, 943 particles have been picked up and kept in an ISO 6 clean room at the Planetary Material Sample Curation Facility of JAXA. Despite a lithology related to LL chondrites that typically have low organic contents, 64 particles from the collection are composed predominantly of carbonaceous material, based on field emission SEM/EDS observation at JAXA Extraterrestrial Sample Curation Center (ESCuC) (Uesugi et al. 2014; Yada et al. 2014b). These carbonaceous particles are classified as “category 3” particles.

**Fig. 10:**
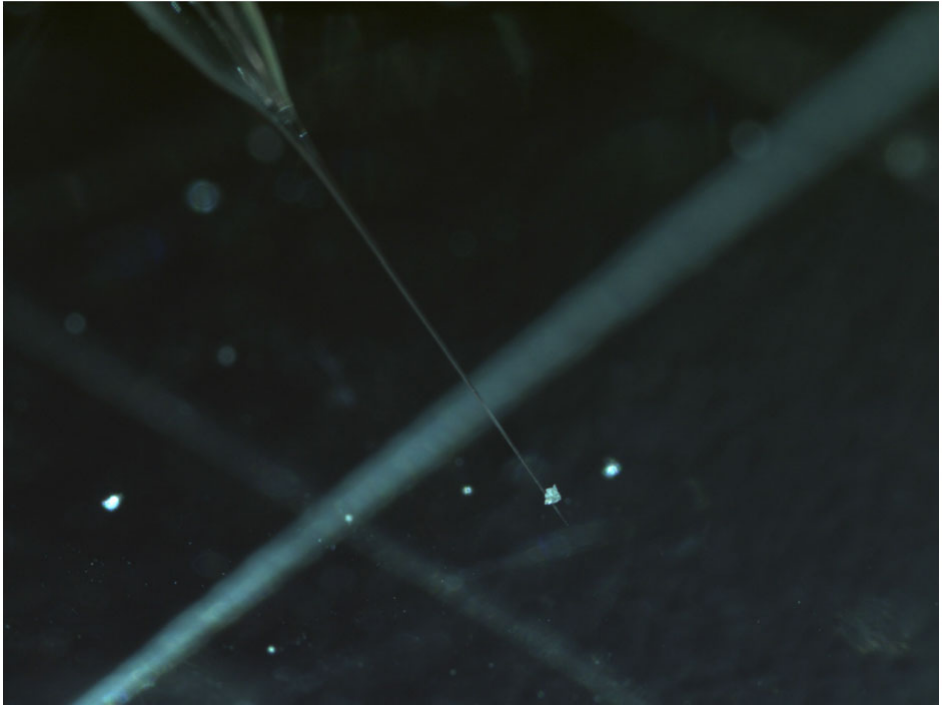
A category 3 particle (RA-QD02-0012) being picked up using a quartz glass needle with platinum wire inside. The size of the particle is about 100 μm. (Image credit: JAXA)

However, the small sizes (∼20–200 μm) of the carbon-rich category 3 particles make the characterisation of their organic contents and determination of their origins (terrestrial vs extra-terrestrial) challenging (Ito et al. 2014; Kitajima et al. 2015; Uesugi et al. 2014; Yabuta et al. 2014a) (Fig. 10). Initial examinations of the category 3 particles indicated that the organic material they contained was not detected on the surface of categories 1 and 2 particles (Naraoka et al. 2012). The particles were composed predominantly of C, N, O and S, and the elemental distributions of the organic material are homogenous (Ito et al. 2014; Naraoka et al. 2015), which make a clear variation from extra-terrestrial IOM that are typically heterogeneous in elemental and isotope distributions (e.g., Aléon et al. 2001; Alexander et al. 2013; Flynn 2008; Quirico et al. 2005). The preliminary results challenged an indigenous origin for the category 3 carbonaceous material because if they were indigenous to the asteroid, at least some of this organic material would have been found as a component of the categories 1 and 2 silicate particles.

Category 3 particles often take different shapes and appearances. Based on the texture of the category 3 particles, they were subcategorised into 3 types: blocky, fibrous and faint (Naraoka et al. 2015; Uesugi et al. 2014). Type 1 blocky particles are the most abundant among the 3 particle types. They are composed of silicate, iron sulphide, stainless steel, and aluminium. They sometimes exhibit rugged edges with “horn-like” texture that is likely produced by tearing. The less abundant category 3 materials are the type 2 fibrous and type 3 faint particles, which are about equal in abundance. However, type 2 fibrous particles have yet to be characterised in any subsequent analysis, so very little is known about their organic composition.

**Fig. 11:**
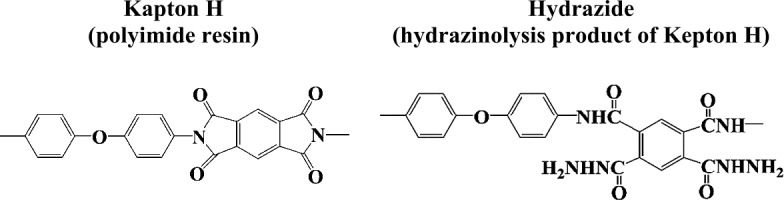
Chemical structures of the sources of the contaminant “white object” (Kitajima et al. 2015)

Subsequent to initial SEM/EDS observations, the organic compositions of selected category 3 particles were further characterised by FTIR, Raman spectroscopy, XANES, ToF-SIMS, and NanoSIMS in order to determine the nature of the organics and their origins. The Raman C parameters of the category 3 particles suggest the presence of disordered organics (i.e. not thermally-altered). However, FTIR spectra of the particles show predominantly C=O stretching with no or very weak aliphatic C–H, which is distinctive from unheated chondritic IOM that often exhibits clear absorption features at around 2960 to 2855 cm^−1^ corresponding to aliphatic C–H in IR spectra (Kitajima et al. 2015). Polyamide – a degradation product of polyimide resin (e.g., derived from polyimide films used in the sample container of the Hayabusa spacecraft) through hydrazinolysis – was suggested to be a possible contaminant that contributed to the C=O, C–F, O–H, and aromatic C=C observed by FTIR for a particle named “white object” (Kitajima et al. 2015; Uesugi et al. 2019; Yabuta et al. 2014b) (Fig. 11).

STXM-XANES analyses of the category 3 particles also came to the same conclusion that the particles displayed typical spectral patterns of heterogeneous organic macromolecules, although they are relatively nitrogen-rich, unlike chondritic IOM (Uesugi et al. 2019; Yabuta et al. 2014a). The results suggested that they are not “fresh/unused” industrial or biological polymers (i.e., *not* fluoro-rubber nor silicon rubber). Instead, they are possibility nitrogen-containing polymer material used in the spacecraft that were degraded through chemical and physical alteration processes (e.g., heat, cosmic ray irradiation) during the 7 years of Hayabusa’s journey, to form the materials seen in these particles. Further XANES investigations have revealed spectral similarity between one of the category 3 particles and the particles collected from a witness plate in a cleanroom where the Hayabusa2 spacecraft was constructed at ISAS/JAXA, indicating contamination into the sample catcher before the operation of the Hayabusa spacecraft (Uesugi et al. 2019).

As the molecular structures and chemical compositions of the category 3 particle organics did not provide a strong indication of an extra-terrestrial/terrestrial origin, further investigation with the use of isotopic analyses were conducted. The refractory organic components in extra-terrestrial materials, such as meteorites, IDPs, and Stardust cometary particles, often contain large D, ^13^C, and ^15^N isotopic enrichments with the presence of hot spots (isotopic anomalies) mixed with moderately enriched isotopically anomalous organic material (e.g., Alexander et al. 2017; Alexander et al. 2007; Busemann et al. 2009; De Gregorio et al. 2013; Messenger et al. 2003; Nakamura-Messenger et al. 2006). However, isotopic analyses of the category 3 particles using NanoSIMS indicated that the organic materials in category 3 particles exhibit terrestrial H, C, and N isotopic compositions, and isotopic anomalies were not observed (Ito et al. 2014; Uesugi et al. 2019). The isotopic compositions of the particles lie in a region that can be represented by either terrestrial or extra-terrestrial organic matter, and since not all extra-terrestrial organic matter contains isotopically anomalous regions, a definitive origin of the category 3 organics could not be established.

The labile organic contents of categories 1 and 2 particles have been explored by Naraoka et al. (2012) using two-dimensional- (2D-) HPLC/FD and ToF-SIMS. Only glycine and alanine were detected in one Itokawa particle at levels slightly higher than that of the procedural blank. As glycine and alanine are common proteic amino acids, while other non-proteic amino acids such as AIB were not detected, Naraoka et al. (2012) concluded that the amino acids present in the samples were likely derived from terrestrial contamination. Similarly, the ToF-SIMS spectra of the Itokawa particles were very similar to that of the procedural blank, with only small differences such as small peaks at $m$/$z = 55$, 57, 73 and 149 in positive ions and $m$/$z = 60$, 61 and 77 in negative ions, which were assigned to phthalates. However, phthalates are likely contaminants introduced to the samples in the laboratory as they are common components of plasticizers.

Witness coupons made from aluminium foil that were exposed in a clean chamber in the curation room at the ESCuC sample curation facility of JAXA were analysed in order to examine the type of contaminants and their accumulation rates (Sugahara et al. 2018). Common terrestrial amino acids (glycine, alanine, valine, leucine, isoleucine, proline, aspartic acid and glutamic acid) were detected. Glycine was the most abundant contaminant with an accumulation rate of 1.2 pmol/cm^2^/day, which could potentially account for the ∼0.1 pmol of glycine detected in the Itokawa particle (Naraoka et al. 2012).

### Extraneous Materials Delivered by Extra-Terrestrial Processes

The process of the delivery of organic matter originating from meteorite infall is not restricted to the planet Earth. Meteorites have been identified on other celestial bodies, such as (i) Bench Crater (McSween 1976; Zolensky et al. 1996) and Hadley Rille (Rubin 1997), which are carbonaceous and enstatite chondrites identified on the Moon, (ii) several iron and stony-iron meteorites (e.g., Meridiani Planum, Lebanon, Littleton) identified on the surface of Mars by Mars Exploration Rovers (MERs) Spirit and Opportunity, and the Curiosity rover of the Mars Science Laboratory (Ashley 2015; Ashley et al. 2011; Connolly et al. 2006; Schröder et al. 2016; Schröder et al. 2008), and (iii) the “Black Boulder”, which is a possible xenolith originates from a low-albedo body identified on Itokawa (Nagaoka et al. 2014). Although the acquisition of exogenous materials is a common process as the target body evolves through time, these ‘foreign’ materials carry information different from the target body and should be carefully identified to avoid confusing their synthetic histories.

Meteoritic impact events can alter the organic records on the asteroidal or planetary body by introducing exogenous organic matter of a different synthetic origin. Impactors are capable of delivering essential exogenous organic molecules (Chyba and Sagan 1992) as well as shock-synthesising organics at the impact zone (Chyba and Sagan 1992; Chyba et al. 1990; Martins et al. 2013). While the shock-synthesised organic molecules are made *in-situ*, organic matter delivered via impact events are synthesised elsewhere and therefore the synthetic process of such organic inventory is unrelated to the local environment. The amount of this exogenous organic is small, for example, only 1–4 wt% of lunar soils was estimated to be derived from carbonaceous chondrite infall, based on the mass balance for iridium in the lunar soils (Haskin and Warren 1991), and approximately 3–6 wt% of carbon at Mercury’s surface (in graphite, amorphous, or nanodiamond form) (Syal et al. 2015). However, the amount of the containing organics is sufficient to introduce complexity to the interpretation of the indigenous organic composition of the lunar soil (Brinton and Bada 1996). For examples, the Bench Crater meteorite was suggested to be the source of complex organic material in the Apollo 17 samples (Thomas-Keprta et al. 2014).

Without a thick atmosphere like that of the Earth, impactors arriving at the surfaces of asteroidal bodies and planets with thin or no atmosphere are not sufficiently decelerated to survive the impact. While small impactors such as IDPs can deliver exogenous organics intact (Anders 1989; Chyba and Sagan 1992; Love and Brownlee 1993), high-energy impact events from larger impactors can alter the organics the impactor contains as well as those present at the surface of the target body. The structures of surviving organic materials can also be altered by short-term impact heating. A group of dehydrated meteorites commonly referred as thermally metamorphosed carbonaceous chondrites have been suggested to have experienced short-term heating induced by transient processes such as impact (Chan et al. 2019c; Nakato et al. 2008; Yabuta et al. 2010). Impact heating may have induced aromatic condensation, dehydrogenation and extensive dehydration of organic molecules, which could have influenced the elemental and isotopic compositions by decreasing the H/C ratio and $\delta $D isotopic value of the organic material (Yabuta et al. 2010). Any traces of alteration prior to the impact event could have been erased by impact heating, which further complicates the determination of the history of organic processing on the parent body.

Other than the extensive cratering record observed for inner and outer solar system planetary bodies (Strom et al. 2005; Zahnle et al. 2003), material mixing through meteorite delivery or asteroidal collision is also supported by meteoritic evidences as xenoliths hosted within another meteorite of a different origin. Primitive xenolithic clasts are found hosted in ordinary chondrites (e.g. Monahans (1998), Zag, Sharps, Carancas, Dar al Gani [DaG] 139), carbonaceous chondrites (e.g. Kaidun), ureilites (e.g. Almahata Sitta, Nilpena, DaG 165), howardite (e.g. Bholghati, EET 87513), and eucrites (LEW 85300) (Brearley and Prinz 1992; Buchanan et al. 1993; Chan et al. 2018a; Chan et al. 2018b; Goodrich et al. 2004; Ikeda et al. 2003; Ikeda et al. 2000; Kebukawa et al. 2017; Rubin et al. 2002; Zolensky and Ivanov 2003; Zolensky et al. 2009; Zolensky et al. 1999; Zolensky et al. 1992). These xenolithic clasts, often referred to as “dark clasts”, contain organic material and mineral components different from the host meteorite (Chan et al. 2018b), and are often classified petrographically, mineralogically, and organically as the primitive types 1 and 2 carbonaceous chondrite matrix-like clasts showing affinities to CM/CI chondrites.

## Contamination Control Measures

A successful sample return mission necessitates the return of scientifically valuable samples unaltered and uncontaminated by the recovery and curation processes. One goal of the Stardust mission was to investigate whether cometary dust contained complex organic materials, and if so, to study the abundance, chemical, and isotopic compositions of the organic phase(s) (Brownlee et al. 2006; Tsou et al. 2003). Hayabusa2 seeks to investigate the “diversification of organic materials through interactions with minerals and water in a planetesimal” (Tachibana et al. 2014). OSIRIS-REx seeks to “return and analyse a sample of pristine carbonaceous asteroid regolith in an amount sufficient to study the nature, history, and distribution of its constituent minerals and organic material” (Dworkin et al. 2017; Lauretta et al. 2017). A proper, carefully planned contamination control is vital in achieving such mission goals, and a realistically and strategically determined level of “acceptable” contamination of the sample is essential to ensure a meticulous and achievable mission outcome (e.g., Dworkin et al. 2017; Sandford et al. 2010; Tachibana et al. 2014; Yada et al. 2014b). This is particularly challenging for organic analysis of returned samples, as the samples are brought to Earth, a planet where water, life and biogenic materials are ubiquitous. With a top priority of returning pristine extra-terrestrial organic matter, any sample returned from space missions must be isolated and protected against any contact with terrestrial contaminants in order to maintain the sample’s pristine nature and scientific importance.

Dworkin et al. (2017) highlighted the essentiality of maintaining the “pristine” state of the returned sample, by underscoring that the “pristine” state can be violated by any alteration to the sample by “changing its inherent states, losing sample components, or adding extraneous components”. One key method to monitor the change to the returned sample’s pristine state is to monitor and evaluate the added terrestrial extraneous component – terrestrial contaminants – which can be achieved by keeping records of the background contamination that can occur at any time during the span of spacecraft fabrication, operations, and sample curation.

Potential sources of terrestrial contaminants should be strictly avoided via preflight, flight, and postflight controls, including any chemical or solvent required for preparing the sampling system or be in contact with the samples or analytical tools, air contained in and material used to build the sample sampler/container, and air circulated in the curation cabinet. Curation of the returned samples (final curation location, sample form, sample catalogue) should be carefully planned to minimise the handling, relocating, or subsampling of the samples, and when subsampling is needed for a particular sample, influence to other samples will be kept to its minimum. Amino acid-based polymers, such as nylon and latex, should be prohibited, and the use of hydrazine should be limited by spacecraft design and operations. Precision cleaning techniques should also be used to remove terrestrial organic and inorganic contamination to a specific level of cleanliness, and such level of cleanliness is verified to a standard. For example, the sample catcher of Hayabusa2 is cleaned in highly purified 2-propanol and methanol/dichloromethane by an ultrasonic cleaner of 38 kHz frequency, which has been subsequently checked by a series of test analyses (Sawada et al. 2017; Yada et al. 2014a). In the past, NASA JSC used Freon 113 as the final cleaning agent for Apollo, and ultrapure water to decontaminate Genesis solar wind materials after hard landing (McCubbin et al. 2019). The use of ultrapure water as final precision cleaning agent is continued nowadays at NASA JSC. Cleaning spacecraft surfaces would require approaches to also limit recontamination and maintain the cleanliness at high levels. The types of organic solvents used to clean the tools and materials should be carefully chosen as they can become the “contaminant” when left as residues to the targeted organic species. For examples, nitriles found in nitrile containing organic solvents or nitrile gloves commonly used in analytical laboratories can potentially be hydrolysed during typical amino acid extraction protocols to give laboratory-yielded amino acids. Subsequent to precision cleaning, all tools which can be baked at high temperatures should be pyrolysed in air at ≥500 °C for at least 3 hours to remove solvent residues and temperature sensitive organic contaminants such as amines, amino, carboxylic, and aromatic acids.

In addition to prudently executing the much-needed contamination control, it is equally important to anticipate the presence of terrestrial contaminants, and to readily identify, monitor, and correct for the organic contamination signatures. Before the return of the samples, the contamination control teams of different missions have made significant research efforts to identify and characterise any intrinsic contaminants in addition to those introduced by the terrestrial environment and sample preparation. To vigorously identify potential contamination sources, “witness coupons”, which were introduced into contamination control protocols for various space missions in the past [e.g. Stardust (Sandford et al. 2010)], have been used to track the origins and routes of incorporation of the contaminants found at each stage of the mission, from the construction of the spacecraft to the curation of the returned samples. The use of witness coupons, in the form of aluminium foil, sapphire glass plates, and aerogel tiles, have been incorporated as a key contamination control procedure in future sample return missions including Hayabusa2, OSIRIS-REx and Mars 2020 (Dworkin et al. 2017; Sawada et al. 2017; Summons et al. 2014; Tachibana et al. 2014).

**Fig. 12:**
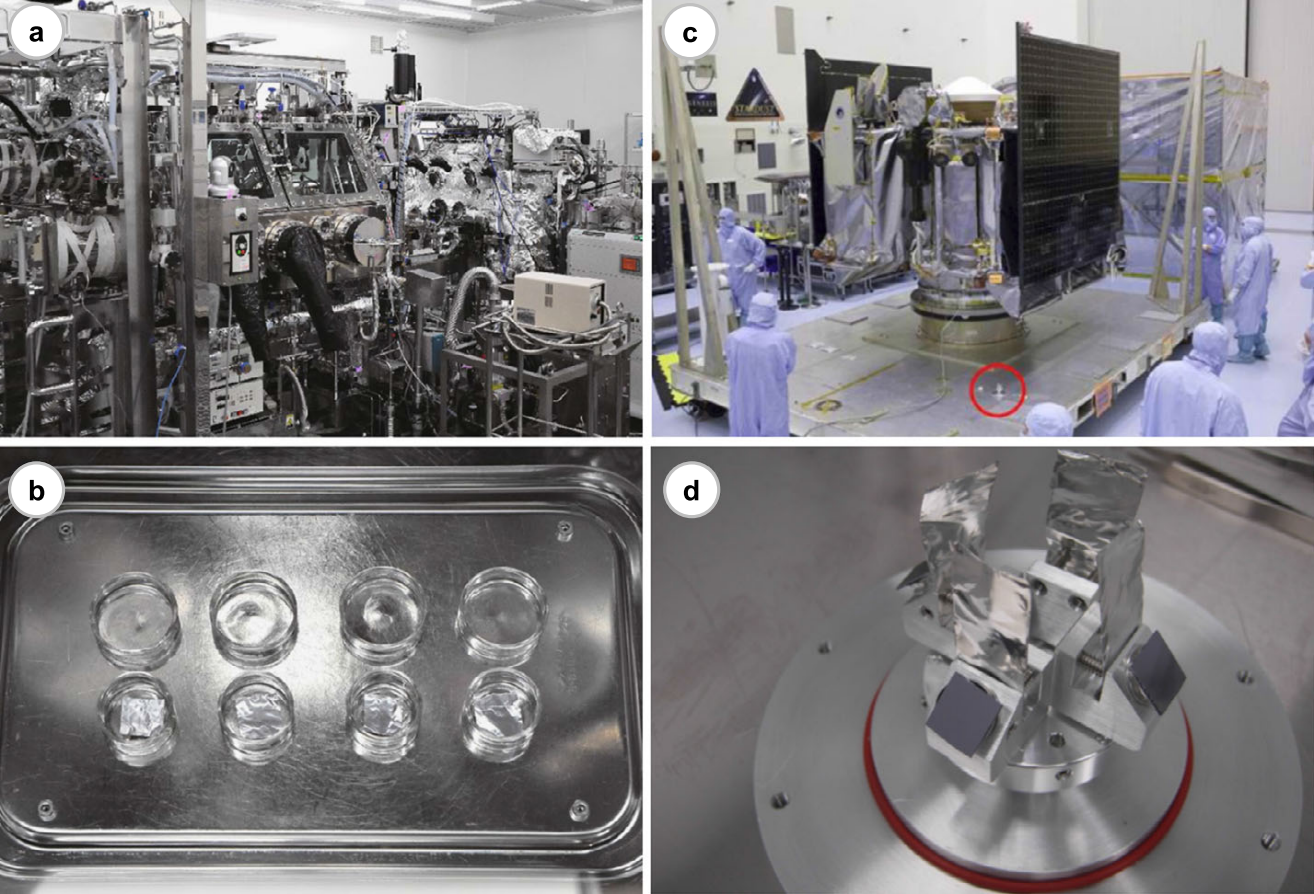
(a, b) The ESCuC sample curation facility of JAXA and the aluminium foil witness coupons (Sugahara et al. 2018).^4^(c, d) Payload Hazardous Servicing Facility of OSIRIS-REx at NASA Kennedy Space Center and the aluminium foil witness plate marked in red circle (Dworkin et al. 2017)^5^

The examination of witness coupons which assessed the level of terrestrial organic contamination in the curation facilities of the Hayabusa2 and OSIRIS-REx missions have been reported recently (Dworkin et al. 2017; Sugahara et al. 2018) (Fig. 12). Seven common proteinogenic amino acids (glycine, alanine, valine, leucine, isoleucine, proline, aspartic acid and glutamic acid) were detected on the witness coupons exposed in the clean chamber in JAXA’s curation room. The total abundance of amino acids detected on the witness coupon that had been exposed to the clean chamber environment for 7 days was 0.3–1.2 ng/cm^2^. The most abundant terrestrial contaminant was glycine, which was accumulated at a calculated rate of 1.2 pmol (∼0.09 ng) /cm^2^/day (Sugahara et al. 2018). This level of terrestrial deposition of glycine is low, compared to the amount of glycine detected in extra-terrestrial samples returned by space missions (e.g. 1.5 ng/cm^2^ detected in the comet-exposed side of the Stardust foil sample (Glavin et al. 2008)) and carbonaceous chondrites (e.g. 2000 ng/g in Murchison (Glavin et al. 2006), <20 ng/g in aqueously and thermally altered meteorites (Burton et al. 2014; Chan et al. 2016)). About 0.05–0.8 ng/cm^2^ of amino acids and <200 cells/cm^2^ of viable cells were detected on the witness coupons exposed to cabinet desiccator boxes located in an ISO 7 clean room at JSC that were reserved for the storage of OSIRIS-REx returned samples (Dworkin et al. 2017). Dworkin et al. (2017) suggested that the total abundance of amino acids could not be explained only by the presence of viable cells on the witness plates and thus the amino acids could have been contributed from other unknown sources. The similar total amino acid abundances observed for witness plates that were exposed to the desiccator boxes for variable lengths of time led the authors to conclude that the total amino acid contamination was not a simple accumulation of material over time. Only very small amounts of volatile compounds were detected at the levels equal to or less than the amounts of volatiles found in the analytical laboratory background.

Witness coupons occupy space and mass on missions, therefore an effective number of witness coupons should be used which are to be strategically placed in key locations (spacecraft construction site, testing environment, on board the spacecraft at multiple positions when flown in space, different locations at the curation facility – e.g., inside curation cabinets, inside the gloveboxes where samples are manipulated, on flow benches where other tools and materials are handled, areas in proximity to air circulation systems to monitor the contaminants from filtered air) and for different target contamination species (Table 1). The recommendations for the use of witness coupons have evolved significantly in recent sample return missions and each mission has a detailed plan of the location, types and numbers of witness coupons used to monitor different types of potential contaminants. For example, Hayabusa2 specified to use (i) thirteen witness coupons (plates, dishes, and disks made of aluminium alloy, Pyrex borosilicate glass, and sapphire glass, respectively, on a base plate) to monitor and record the environment during manufacturing of the sampler, its installation to the spacecraft and transportation between facilities including the launch site, (ii) nine coupons on the sampler, and (iii) two inside the sample catcher and container to monitor the inner environment of the entire mission (Sawada et al. 2017). OSIRIS-REx specified that two types of witness plates (sapphire and aluminium) should be flown – ten flown in the sample return capsule and fourteen on the touch-and-go sample acquisition mechanism (TAGSAM), among a well-documented list of other types of coupons used for background facility contaminants and hardware verification (Dworkin et al. 2017).

In addition to the careful plans to monitor the background contamination level from as early as the manufacture of the spacecraft, the flow of mission returned sample analysis should take into consideration that any contaminant accrued by a technique does not influence the interpretation of the data collected by subsequent analytical techniques. Although a coordinated analysis involving multiple analytical techniques can provide a better picture of the organic content of the valuable mission returned samples, the number and types of analytical techniques used in an analytical sequence should be carefully considered, not only to reduce the organic alteration but also to limit the exposure of the sample to potential contaminants. The analytical sequence should always prioritise non- or less-destructive techniques (Table 3). For example, for refractory organic analysis, FTIR should ideally be placed prior to Raman and UV fluorescence spectroscopy, as the organics can be thermally damaged and photo-oxidised by the laser used in the later techniques if the laser intensity is set too high. L^2^MS can be used to detect aromatic/conjugated organic molecules at the micrometre scale and the sub-attomole level. However, this technique is considered mildly destructive as it desorbs molecules from the sample surface, and therefore should be conducted subsequent to Raman spectroscopy. SEM is commonly conducted at the beginning of an analytical sequence as it provides images/elemental maps of a sample that help to determine areas of interest. However, SEM also can introduce C contamination during electron irradiation in particular when imaging at high magnifications. The contamination is a function of the accelerating voltage (C ∝ V^−0.8^) and current density, duration of beam exposure, conductivity of the sample, and the presence of hydrocarbons, which could reduce the image contrast, and impose artefacts on subsequent C analysis by depositing a layer of disordered C (Chan et al. 2019b). Therefore, it is recommended to place SEM analysis in a later sequence if that is permissible, or verification of the cleanliness of the SEM chamber through high resolution imaging of pre-cleaned metal stubs. STXM-XANES allows chemical mapping (with a high spatial resolution of 30 to 40 nm) and can produce spectra that reveal considerable chemical complexity in the organic macromolecules. However, if focussed ion beam (FIB) extraction of a section is required for the XANES analysis, rather than ultramicrotome sectioning, the sample can suffer from extensive thermal and irradiation damage (Bassim et al. 2012). Finally, SIMS techniques shall be the last of the experimental sequence due to the destructive nature of the ionisation process.

**Table 3:**
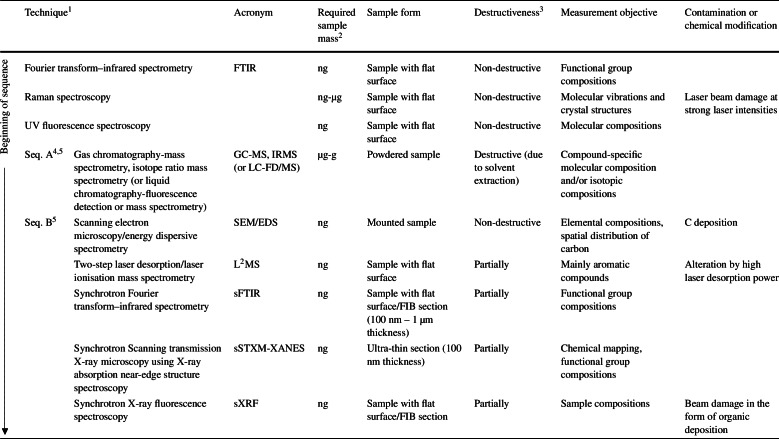

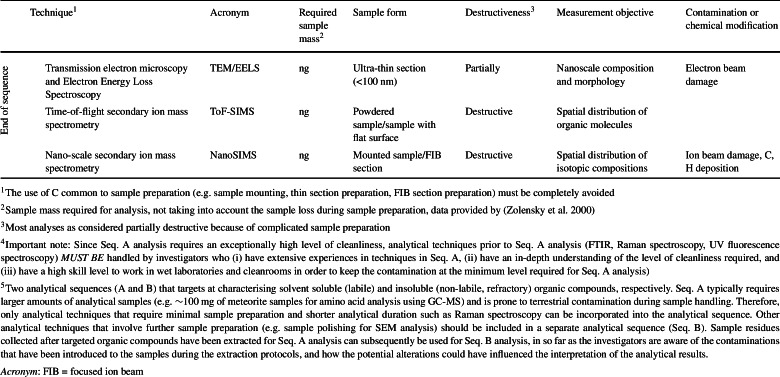
A list of the most common analytical techniques used for characterisation of organic components in extra-terrestrial samples and the suggested analytical sequence

Labile organic analysis often requires the extraction of the target molecules and thus the technique is highly destructive. After the labile organic extraction process, sample residue and refractory materials can be collected, however, they are often extensively altered during the course of the extraction procedure. Therefore, it would be more sensible to allocate samples separately for labile and refractory organic analyses. However, if the availability of samples is extremely limited, labile organic extraction can be performed after the same sample is characterised by a few non-destructive analytical techniques, such as FTIR and Raman spectroscopy. Very often, the contamination controls taken during labile and refractory organic analyses are different. For examples, samples targeted for amino acid analysis can only be in contact with sterile materials, or tools that have been sterilised after subjecting to heating at 500°C in air for at least 3 hours, while samples analysed by FTIR and Raman spectroscopy can be in contact with tools that have only been cleaned with organic solvent. If a sample is targeted for amino acid analysis subsequent to characterisation using FTIR and Raman spectroscopy, the same level of cleanliness should be sought for during the entire analytical sequence.

## Conclusion

Sample return missions are essential in order for us to answer fundamental questions regarding the nature of the organic matter at birth of our solar system, its subsequent evolution, and the implications on the origin of life on Earth. However, proper identification and characterisation of extra-terrestrial organic matter are complicated by potential organic contamination, as the samples are exposed to terrestrial environment subsequent to their arrival on Earth, a planet inhabited by life. Unfortunately, the samples will never be cleaner than the tools and containers used store and handle them. A key lesson learned from past sample return missions is that a certain level of terrestrial contamination is inevitable, despite the best efforts that were made to minimise it. While careful measures of contamination control are planned and implemented, future studies of mission returned samples should be aware of the presence of different levels of terrestrial contamination, and employ state-of-the-art methods in order to distinguish extra-terrestrial organics form the inevitable terrestrial contamination.

## Footnotes

1 Reprinted from Geochimica et Cosmochimica Acta, 172, Jamie E. Elsila, Michael P. Callahan, Jason P. Dworkin, Daniel P. Glavin, Hannah L. McLain, Sarah K. Noble, Everett K. Gibson, The origin of amino acids in lunar regolith samples, 357-369, 2016, with permission from Elsevier.

2 Reprinted from Chemie der Erde - Geochemistry, 71, Carlton Allen, Judith Allton, Gary Lofgren, Kevin Righter, Michael Zolensky, Curating NASA’s extraterrestrial samples—Past, present, and future, 1-20, (2011), with permission from Elsevier.

3 Reprinted from Meteoritics & Planetary Science, 49, David R. Frank, Andrew J. Westphal, Michael E. Zolensky, et al., Stardust Interstellar Preliminary Examination II: Curating the interstellar dust collector, picokeystones, and sources of impact tracks, 1522–1547, (2013), with permission from John Wiley and Sons.

4 Reprinted from Earth, Planets and Space, 70, Haruna Sugahara, Yoshinori Takano, Yuzuru Karouji, Kazuya Kumagai, Toru Yada, Naohiko Ohkouchi, Masanao Abe and Hayabusa2 project team, Amino acids on witness coupons collected from the ISAS/JAXA curation facility for the assessment and quality control of the Hayabusa2 sampling procedure, 1–10 (2018). This article is distributed under the terms of the Creative Commons Attribution 4.0 International License (http://creativecommons.org/licenses/by/4.0/).

5 Reprinted by permission from Springer Nature, Space Science Reviews, OSIRIS-REx Contamination Control Strategy and Implementation, J. P. Dworkin, L. A. Adelman, T. Ajluni et al. (2017).
